# Supplementary material for: Content-rich biological network constructed by mining PubMed abstracts
Source: BMC Bioinformatics. 2004 Oct 8;5:147. doi: 10.1186/1471-2105-5-147 (PMC528731; doi:10.1186/1471-2105-5-147)
Supplement: Additional File 2 — The original results of the above study (non-essential files are deleted to keep the file size under the limit set by BMC bioinformatics). [file 1471-2105-5-147-S2.bz2 › chilibotAdditionalFile2/dip30/5ID9184158E14/html/TCF3_MYOD1.html]

 


 **TCF3** and **MYOD1** 
  
Found 43 abstracts in PubMed, retrieved 30.  
 

 What does Google say? 
 PDF only 
| .edu only 

---

**Interactive relationship** (e.g. stimulation, inhibition, etc)

**Neutral relationship**- Here, we report that Pbx Meis1 Prep1 binds DNA cooperatively with heterodimers of E2a  [ **TCF3** ]  and MyoD  [ **MYOD1** ] , myogenin, Mrf 4 or Myf 5.  Ref: 10471746 Nucleic Acids Res, 1999
- Furthermore, we found that MyoD  [ **MYOD1** ]  co transfected with either ITF  [ **TCF3** ]  2B or ITF  [ **TCF3** ]  2A converted fibroblasts into myoblasts with the same frequency.  Ref: 10833525 J Biol Chem, 2000
- MyoD  [ **MYOD1** ]  heterodimerizes with E type factors E12 E47 and ITF  [ **TCF3** ]  2A ITF  [ **TCF3** ]  2B and binds E box sequences within promoters of muscle specific genes.  Ref: 10833525 J Biol Chem, 2000
- Here, we examine the interactions of full length MyoD  [ **MYOD1** ]  with itself and with an HLH containing peptide portion of an E2A  [ **TCF3** ]  gene product, E47 96.  Ref: 8524857 Proc Natl Acad Sci U S A, 1995
- These E2A  [ **TCF3** ]  monomers cannot bind DNA but heterodimerize efficiently with Id and MyoD  [ **MYOD1** ] .  Ref: 8001133 Cell, 1994
- In transient transfection assays, MyoD  [ **MYOD1** ]  activates transcription in the presence of ITF  [ **TCF3** ]  2A but not ITF  [ **TCF3** ]  2B, which contains a 182 amino acid N terminal extension.  Ref: 10833525 J Biol Chem, 2000
- Thus, differentially spliced transcripts of mouse ITF  [ **TCF3** ]  2 encode different proteins that appear to dimerize with MyoD  [ **MYOD1** ]  and activate or repress transcription.  Ref: 8631961 J Biol Chem, 1996
- The myoD  [ **MYOD1** ]  protein was present in the nucleus in cells grown as attached or aggregated cultures and, in both culture conditions, the myoD  [ **MYOD1** ]  protein was associated with transcription factors of the E2A  [ **TCF3** ]  family and was able to bind DNA at E box sequences.  Ref: 10438573 Exp Cell Res, 1999
- In muscle, E2A  [ **TCF3** ]  proteins bind DNA as heterodimers with muscle specific bHLH partners, such as MyoD  [ **MYOD1** ]  and myogenin, and these heterodimers are thought to be both necessary and sufficient for muscle determination in cultured cells.  Ref: 7623842 Mol Cell Biol, 1995
- In many instances tissue specific basic helix loop helix proteins, such as the hematopoietic factor SCL tal or the myogenic factor MyoD  [ **MYOD1** ] , interact with ubiquitously expressed basic helix loop helix proteins, such as E2A  [ **TCF3** ]  or E2 2.  Ref: 8576241 J Biol Chem, 1996
- ITF  [ **TCF3** ]  2 is a bHLH protein with widespread expression that is thought to form active heterodimers with MyoD  [ **MYOD1** ] , a muscle specific bHLH transcription factor.  Ref: 8631961 J Biol Chem, 1996
- A splice variant of the ITF  [ **TCF3** ]  2 transcript encodes a transcription factor that inhibits MyoD  [ **MYOD1** ]  activity.  Ref: 8631961 J Biol Chem, 1996
- As expected, overexpression of either Id3 HLH462 or ITF  [ **TCF3** ]  2b effectively inhibited the activation of the muscle specific creatine kinase promoter by the myogenic transcription factor MyoD  [ **MYOD1** ] .  Ref: 8999959 J Biol Chem, 1997

**Non-interactive relationship** (e.g. studied together, co-existance, homology, etc.)

- We have investigated whether the myogenic residues influence DNA sequence recognition and how MyoD  [ **MYOD1** ] , Twist, and their E2A  [ **TCF3** ]  partner proteins prefer distinct CAN NTG sites.  Ref: 10594029 Mol Cell Biol, 2000
- The observation that the bHLH domain is present in the AH receptor and the ARNT protein, a factor required for proper AH receptor function, suggests that these proteins are heterodimeric partners that activate gene expression in a manner similar to Myc Max and MyoD  [ **MYOD1** ]  E2A  [ **TCF3** ] .  Ref: 8287061 Pharmacogenetics, 1993
- The box I and II motif located within the promoter of the myelin basic protein gene contains a simian virus 40 T antigen binding site, a MyoD  [ **MYOD1** ]  E2a  [ **TCF3** ]  binding site, and a glucocorticoid receptor binding site.  Ref: 7531995 J Mol Neurosci< MedlineTA>J Mol Neurosci, 1993
- An E box binding activity, which does not contain MyoD  [ **MYOD1** ] , myogenin, or E2A  [ **TCF3** ]  proteins, is present in muscle and nonmuscle cells and may be responsible for repressing the delta subunit gene in myoblasts and nonmuscle cells.  Ref: 8355673 Mol Cell Biol, 1993
- The myogenic regulatory factors MRFs, MyoD  [ **MYOD1** ] , myogenin, Myf 5, and MRF4, function as transcriptional activators of muscle specific gene expression by forming heterodimers with the ubiquitously expressed products of the E2A  [ **TCF3** ]  gene, E12 and E47.  Ref: 9116507 Protein Expr Purif, 1997
- Analysis of the inhibition of MyoD  [ **MYOD1** ]  activity by ITF  [ **TCF3** ]  2B and full length E12 E47.  Ref: 10833525 J Biol Chem, 2000
- mRNA transcripts for E2A  [ **TCF3** ]  gene products were also expressed in mesangial cells, but these cells failed to express mRNAs for MyoA MyoD  [ **MYOD1** ]  related genes.  Ref: 8284227 Nucleic Acids Res, 1993
- In these cells, which displayed unchanged levels of the ubiquitous basic helix loop helix E2A  [ **TCF3** ]  factors and Id proteins, Tax was found to target the muscle specific basic helix loop helix transcription factor MyoD  [ **MYOD1** ] .  Ref: 11751456 Cell Growth Differ, 2001
- In NIH3T3 fibroblasts, the ubiquitous helix loop helix HLH protein E2A  [ **TCF3** ]  E12 E47 and the myogenic HLH proteins MyoD  [ **MYOD1** ] , MRF4 and myogenin are growth inhibitory, while two ubiquitous Id proteins lacking the basic region are not.  Ref: 7925274 EMBO J, 1994
- Mutation of this E box, which prevents binding of MyoD  [ **MYOD1** ]  E2A  [ **TCF3** ]  and myogenin E2A  [ **TCF3** ]  heterodimers, decreases expression in myotubes and increases expression in myoblasts and nonmuscle cells.  Ref: 8355673 Mol Cell Biol, 1993
- In addition, mechanisms that position the BR allow E2A  [ **TCF3** ]  to prefer distinct half sites as a heterodimer with MyoD  [ **MYOD1** ]  or Twist, indicating that the E2A  [ **TCF3** ]  BR can be directed toward different targets by dimerization with different partners.  Ref: 10594029 Mol Cell Biol, 2000
- E2A  [ **TCF3** ]  505 513 interacted with mUbc9 but not with human Ubc5, MyoD  [ **MYOD1** ] , Id3, or the polymyositis scleroderma autoantigen.  Ref: 10497239 J Biol Chem, 1999
- Further investigation indicated that the ITF  [ **TCF3** ]  2b MyoD  [ **MYOD1** ]  heterodimer bound to its specific DNA binding site in vitro, and the DNA binding was effectively blocked by Id3 HLH462.  Ref: 8999959 J Biol Chem, 1997
